# Knowledge and Perceptions Regarding Coronavirus (COVID-19) among Pediatric Dentists during Lockdown Period

**DOI:** 10.3390/ijerph19010209

**Published:** 2021-12-25

**Authors:** Sreekanth Kumar Mallineni, Sivakumar Nuvvula, Jaya Chandra Bhumireddy, Ahmad Faisal Ismail, Priya Verma, Rishitha Sajja, Abdullah Alassaf, Basim Almulhim, Sara Alghamdi, Anupam Saha, Virinder Goyal, Srinivas Namineni

**Affiliations:** 1 Department of Preventive Dental Science, College of Dentistry, Majmaah University, Al Majma’ah 11952, Saudi Arabia; am.assaf@mu.edu.sa (A.A.); B.almulhim@mu.edu.sa (B.A.); Sa.mohamed@mu.edu.sa (S.A.); 2 Center for Transdisciplinary Research (CFTR), Saveetha Institute of Medical and Technical Sciences, Saveetha Dental College, Saveetha University, Chennai 600077, Tamil Nadu, India; 3 Department of Pediatric and Preventive Dentistry, Narayana Dental College and Hospital, Nellore 524003, Andhra Pradesh, India; dentist4kids@gmail.com; 4 Department of Pediatric and Preventive Dentistry, Saraswati Dhanwantri Dental College and Hospital & Post Graduate Research Institute, Parbhani 431401, Maharashtra, India; dr.jayachandra2509@gmail.com; 5 Department of Paediatric Dentistry and Dental Public Health, Kulliyyah of Dentistry, International Islamic University Malaysia, Gombak 25200, Selangor, Malaysia; drfaisal@iium.edu.my; 6 Department of Pediatric and Preventive Dentistry, Bhabha Institute of Dental Sciences, Bhopal 462026, Madhya Pradesh, India; priya.verma@live.co.uk; 7 Bristol Myers Squibb, Pennington, NJ 07922, USA; sajjarishi88@gmail.com; 8 Pediatric Dentistry, Sairam Dental Hospital, Calcutta 700012, West Bengal, India; saha5976@gmail.com; 9 Department of Pediatric and Preventive Dentistry, Gurunanak Dev Dental College, Patiala 148028, Punjab, India; virinderg@gmail.com; 10 Pediatric Dentistry, Rainbow Hospitals, Hyderabad 500034, Telangana, India; namineni.srinivas@gmail.com

**Keywords:** coronavirus, COVID-19, dentistry, knowledge, N95, Pediatric Dentistry, perceptions

## Abstract

Aim: To assess the knowledge and perceptions of COVID-19 among pediatric dentists based on their dependent source of information. Methods: A descriptive-analytical cross-sectional survey using a self-administered questionnaire with 23 questions was sent via Google forms to pediatric dentists. All participants were divided into three groups [postgraduate residents (PGs), private practitioners (PP), and faculty (F)]. The comparison of knowledge and perception scores was made based on occupation, source of information, and descriptive statistics used for the analysis using SPSS 21.0 (IBM, Armonk, NY, USA). Results: A total of 291 pediatric dentists completed the survey, and the majority of them were females (65%). Overall, good mean scores were obtained for knowledge (9.2 ± 1.07) and perceptions (5.6 ± 1.5). The majority of the participants used health authorities (45%) to obtain updates on COVID-19, while social media (35.1%) and both (19.6%) accounted for the next two. A statistically significant difference (*p* < 0.05) was found among different pediatric dentists groups for relying on the source of information. Conclusion: Overall good pediatric dentists showed sufficient knowledge regarding COVID-19. The pediatric dentists’ age, occupation, and source of information influenced knowledge regarding COVID-19, whereas perceptions were influenced by age and gender of the participants. Health authorities successfully educated pediatric dentists than the social media

## 1. Introduction

The recent pandemic outbreak of COVID-19 disease has posed significant healthcare industry challenges. Dentists are at a higher risk for contracting COVID-19 illness among all the professionals dealing with the population daily. It could be due to: (i) the close contact of the patient to the dentist; (ii) proximity to the work area and nasal orifices; and (iii) the production of contaminated aerosol that stays suspended in the air for quite some time. These reasons make the dental profession difficult during this pandemic outbreak of COVID-19 disease. Subsequently, the provision of all routine dental procedures has been suspended in most parts of the world [1]. Meng and co-workers [2] reported nine positive cases among 169 oral health care workers due to aerosol inhalation and contamination with blood and saliva. The first case of a dentist who tested positive for COVID-19 was reported from a dental Hospital, Wuhan University, China. Eventually, the disease was transmitted to eight other dental professionals [2,3]. Chan and co-workers [4] reported direct contact transmission as potentially high risk for COVID-19, such as contaminated hands that could facilitate transmission.

Although, based on existing finings COVID-19 effected children are asymptomatic and most infected children may not fall sick compared to adults [5]. Pediatric dentists are the one who deals with dental problems in children [6]. In most dental procedures, pediatric dentists appear to be at a higher risk due to proximity with child oral cavities that cause exposure to blood, saliva, or aerosol production [6,7]. This infected aerosol can remain suspended in the air for quite some time. It can act as a potential infection source that can pass through inhalation of droplets or by directly contacting the contaminated surface and instruments [8]. To avoid the potential transmission of COVID-19 in the pediatric dental operatory, it has become crucial to know whether pediatric dentists know enough about COVID-19 diseases and their new occupational hazards. The source of information regarding COVID-19 is essential in creating awareness among healthcare professionals. The health authorities, such as the World Health Organization (WHO), Centre for Disease Control and Prevention (CDC), American Dental Association (ADA), Johns Hopkins Corona Research Centre (JHCRC), and local health regulatory bodies of different countries are providing daily updates on COVID-19. With the high volume of information reaching us every day through various sources, the dynamics of COVID-19 are evolving rapidly. Social media (Twitter, Facebook, Instagram, WhatsApp, and YouTube) also became a significant source of information for daily updates on COVID-19 [1]. It is essential to check the influence of data sources on the knowledge and perceptions of COVID-19 disease among pediatric dentists. Hence, the present study aimed (i) to assess the knowledge and perceptions of pediatric dentists during the COVID-19 pandemic outbreak and (ii) evaluate the knowledge and perceptions of COVID-19 among pediatric dentists based on their dependent source of information.

## 2. Materials and Methods

A descriptive-analytical cross-sectional design was used with a convenience sampling technique for this study. This qualitative study assessed knowledge and perceptions of the current COVID-19 pandemic outbreak among pediatric dentists. A questionnaire was used to determine the knowledge and perceptions, consisting of various questions. A research team assessed the content validity of the questionnaire used in the study. This cross-sectional survey was piloted in March 2020. This pilot study was conducted to measure the reliability of the tool used in the study. A welcome message was sent along with the Google form link regarding the study information, and only participants with willfulness upon acceptance are allowed to respond to the questionnaire. One response was locked to one device to avoid multiple responses from one individual. The questionnaire (Supplemental data) covered comprehensive knowledge and perception regarding COVID-19 that was beneficial in achieving the objectives of the study. The convenience sample was used in the study, and the questionnaire (23 questions) was sent to WhatsApp groups (PedoBuddies, Pediatric Dentistry Club, and South Asian Association Pediatric Dentistry) via Google forms. Responses from dentists other than pediatric dentists and incomplete questionnaires were excluded from the analysis. Knowledge was assessed through questions centering on COVID-19 etiology, signs, and symptoms. The study was approved by the institutional ethical committee, Majmaah University, Saudi Arabia, under the No MURE-July22/COM-2020/36-1. Written informed consent for participation was not required for this study.

### Statistical Analysis

Data were analyzed using SPSS version 21.0 (IBM Corp, Version 21.0. Armonk, NY, USA). Means and standard deviations were used to describe the continuous variables, and percentages were used to describe the categorical data. Mean values of knowledge and perceptions were including transmission, and risk prevention. Each response was scored as “1” (correct) and “0” (wrong), with scores ranging from 1 to 10 for knowledge and 1 to 8 for perceptions. A cut-off level was considered median to indicate poor knowledge about COVID-19, whereas scores above the median (>median) were deemed adequate knowledge about COVID-19 disease. Perceptions were assessed by focusing questions on the awareness and attitudes of pediatric dentists on COVID-19. The pediatric dentists’ perceptions were categorized as low (score ≤ median) or good (>median). All the participants were divided into three groups based on their occupation [postgraduate residents (PG), faculty (F), and private practitioners (PP)]. In addition to the questionnaire, all the participants were asked whether they had sufficient knowledge about COVID-19. The Chi-square test used to compare binary data and comparison among the groups has been analyzed using ANOVA with multiple comparisons tests Bonferroni correction with a 95% confidence interval (*p* < 0.05). The multivariate linear regression analysis used the association between demographic variables and the pediatric dentists’ knowledge and perception scores. The multivariate linear regression analysis used the association between demographic variables and the pediatric dentists’ knowledge and perception scores. For the regression analysis, female (gender), postgraduate nets (occupation), and social media (Source of information) were used as reference points for comparison.

## 3. Results

**Figure 1:**
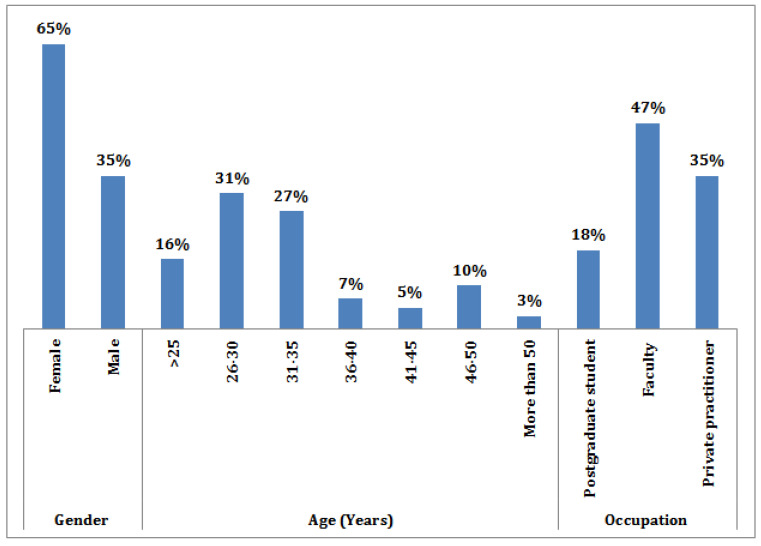
Demographic characteristics of the study population.

**Figure 2:**
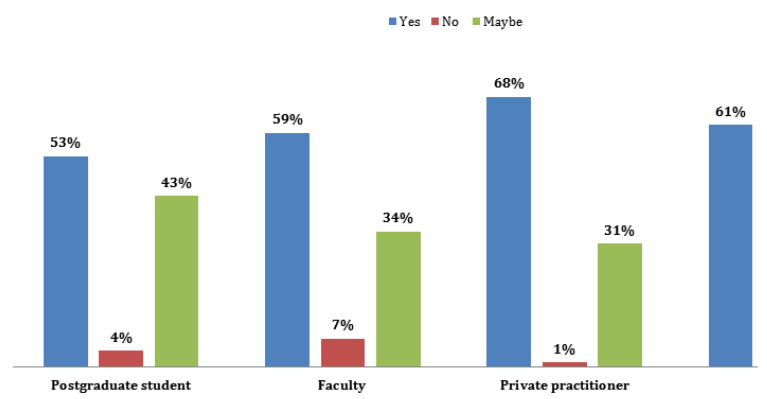
Responses for the question on their perception of sufficient knowledge on COVID-19.

A total of 291 pediatric dentists completed the survey, and the majority were females, 65% (*n* = 188), while males were 35% (*n* = 103). Pediatric dentists of varying ages from 25 years to more than 50 years participated in the study. Most of the responses for the survey were obtained from faculty [F] (46.7%), followed by private practitioners [PP] (35.4%) and postgraduate residents [PG] (17.9%) (Figure 1). The questionnaire was sent to three WhatsApp groups, 300 participants responded. Nine participants (3%) were excluded from the study due to the incomplete submission of questionnaires. Overall knowledge scores (91.4) and perceptions scores (56.4%) achieved by pediatric dentists participated in the study with mean scores of 9.2 ± 1.07 and 5.6 ± 1.5, respectively. Overall, 61% of the pediatric dentists communicated that they have sufficient knowledge of COVID-19, while 34% of the pediatric dentists expressed doubt, and 5% reported insufficient knowledge (Figure 2). Among all the participants, private practitioners achieved higher scores (68%), followed by faculty (59%), and postgraduate residents (53%).

Source of knowledge about COVID-19 disease was through media (35.1%), health authorities (45.4%), and both (19.6%). Social media was a significant source of information, whereas the health authorities were considered as the primary source amid health authorities. A statistically significant difference (*p* < 0.05) was found among different pediatric dentists, relying on the source of information (Table 1). Faculty members significantly outweighed other groups in describing health authorities as reliable sources, whereas private practitioners believed both media and health authorities were reliable sources. A statistically significant *p*-value (*p* < 0.05) was found on statistical analysis using the ANOVA test regarding the source of information. In the association between demographic variables and the pediatric dentists’ knowledge scores using the multivariate linear regression analysis, a significant difference was found regarding the source of knowledge about COVID-19 disease (Table 2).

**Figure 3:**
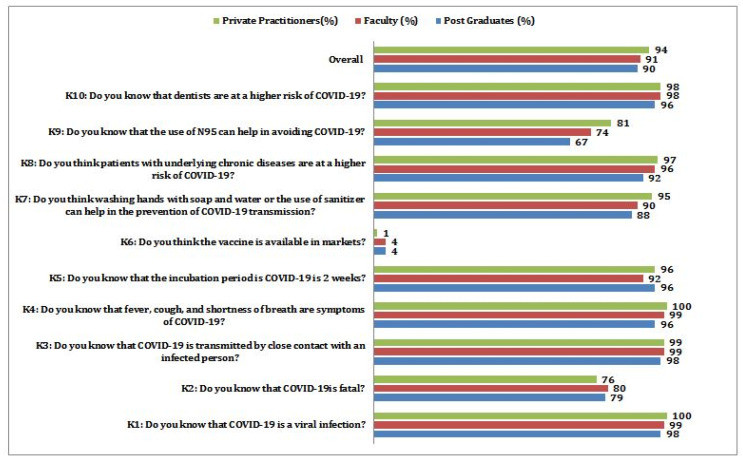
Overall knowledge scores (%) of pediatric dentists participated in the study.

Overall, the range of knowledge scores achieved was 2–10, with a mean of 9.2 + 1.07 (Table 3). Good knowledge scores were obtained from the 82.5% study population, while low knowledge scores were obtained from 17.5% (*p* < 0.05). Overall good scores were obtained for all the three groups (PG-89.5%, F-91.4%, and PP-93.6%) participating in the study, respectively (Figure 3). Most of the participants had good knowledge about COVID-19 disease as a viral infection (PG-98%, F-98%, and PP-100%), and most of them were aware that a severe fatality could result from COVID-19 (PG-79%, F-80%, and PP-76%). Almost all participants knew that the virus could transmit from person to person by close contact (PG-98%, F-98%, and PP-99%). Although a few were not familiar with the common symptoms of COVID-19, the majority had a good knowledge of flu-like symptoms (PG-96%, F-98%, and PP-100%).

**Figure 4:**
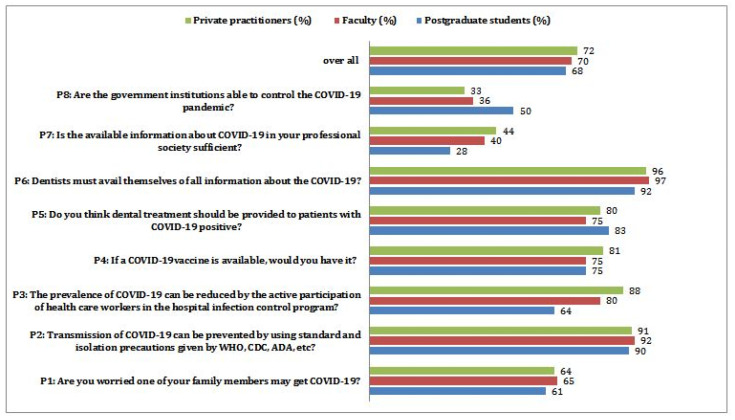
Overall Perception scores (%) of pediatric dentists participated in the study.

A majority of the participants knew that the incubation period of COVID-19 disease is two weeks (PG-96%, F-92%, and PP-96%), and 91% of the participants felt that vaccine is not available for COVID-19 disease (PG-85%, F-91%, and PP-94%) among these, a minority of postgraduates (15%) had a lack of awareness. More than 90% of respondents showed good knowledge of preventive measures, such as frequent hand washing, sanitizer (PG-88%, F-89%, and PP-95%). The knowledge of an increased risk of the infection in chronic disease patients seems to be underrated (PG-92%, F-96%, and PP-97%) compared to other questions. Only 75% of participants knew that N-95 mask use could avoid transmission of COVID-19 disease (PG-67%, F-74%, and PP-81%), while postgraduate residents had shallow knowledge about usage of N-95 masks. A majority (98%) of participants felt that dentists are at a higher risk of COVID-19 disease in the dental operatory (PG 92%, F 98%, and PP 98%) (Figure 3). Only 64% of participants were worried that COVID-19 diseases might transmit to their family members (PG-61%, F-65%, and PP-64%) (Figure 4). The majority of the participants (91%) believed that the transmission could be halted by following standard guidelines given by WHO, CDC, ADA, etc., (PG-90%, F-92%, and PP-91%), but the responses towards the hospital infection control program to reduce COVID-19 disease varied among the groups (PG-64%, F-80%, and PP-88%). The willingness to have a vaccine against this fatal disease was supported by 77% of the pediatric dentists (PG-75%, F-75%, and PP-81%). Only 78% of the pediatric dentists showed a willingness to provide dental treatments to COVID-19 positive children (PG-83%, F-75%, and PP-80%). There was a remarkable response to the attitude of availing updated information themselves about COVID-19 (PG-92%, F-97%, and PP-96%). Very few participants believed that presently available information about COVID-19 is sufficient (PG-28%, F-40%, and PP-44%), and 60% of the pediatric dentists felt professional societies should provide enough information. Only 38% of pediatric dentists opined that government institutions could control this pandemic outbreak (PG-50%, F-36%, and PP-33%). The variance analysis was applied to know the effect of information sources on knowledge scores among various groups of pediatric dentists. A statistically significant difference (*p* < 0.001) was observed among participants who followed the media as a significant information source with a mean of 8.8 and SD +/−1.41. When multiple comparisons were applied, the combined influence of media and health authorities made a statistically significant difference (*p* < 0.05). The minimum and maximum scores for knowledge are 2 and 10, with a mean of 9.2 and a standard deviation of ±1.07. With a cut-off score of 8 being graded as good, 82.5% achieved high/good knowledge scores, and 17.5% obtained low/poor knowledge scores (Table 3). The overall inference was that most participants received good knowledge from various information sources. The multivariate regression analysis showed that participants’ age, occupation, and utilized source of formation have a significant effect on knowledge (*p* < 0.05), whereas age does not found to have any impact on the knowledge (*p* > 005) (Table 4). The multivariate regression analysis showed that participants’ occupation (faculty) and source of information has no significant effect on the perceptions (*p* > 0.05) (Table 4). However, study participants age, gender, and occupation (private practitioners) found to have significant co-relation with percept (*p* < 0.05).

## 4. Discussion

The role of the dentist can be highly contributory in the pandemic outbreak by detecting the initial symptoms, supporting the population as a medical adjunct during the time of emergency, working and maintaining a safe clinical environment for themselves and patients, and reporting any suspected patient while performing a thorough evaluation in terms of current health status. The present cross-sectional study reports the knowledge and perceptions of the COVID-19 outbreak among pediatric dentists. Questionnaire-based studies have been proven as an effective data collection method to gather information [9]. The participants were divided into three categories: postgraduate residents [PG], faculty [F], and private practitioners [PP] for analysis purposes. However, there are some surveys among dentists and have been published [1,10,11,12,13,14,15]. In the present study, pediatric dentists were postgraduate residents (18%), private practitioners (35%), and faculty (47%). Similarly, all participants were orthodontists in a study among Italian dentists [12], whereas 16.8% were orthodontists, 12% were oral surgeons, and 2% were pediatric dentists in another study from Italy [13]. In contrast, Gambir et al. [15] performed an analysis with all private practitioners in India. Duruk et al. [1] survey included only 65.1%, general dentists. In a Jordanian study, 60% of participants were private practitioners [11] and while 74% of participants were private practitioners in a study among 30 countries [9]. A study in Saudi Arabia had dental interns, auxiliaries, and specialist dentists as participants [16]. In the present study, entire participants were pediatric dentists. In a Turkey study, 10.3% were pediatric dentists [1], whereas 1.7% participated in Italy’s study [13].

The knowledge-based questions were related to the signs and symptoms, transmission, and prevention of COVID-19. Based on the overall knowledge scores obtained, it is inferred that all the participants had good knowledge of COVID-19 disease. Private practitioners achieved higher knowledge scores (93.6%) compared to faculty (91.4%) and PGs (89.5%). A recent systematic review [17] reported that the overall knowledge scores were 79% which is low compared to the score in the present study (91.5%). The majority of the participants (PGs: 96%, F: 98%, PP: 98%) believe that the N95 mask can avoid transmitting the disease, indicating pediatric dentists in the present study had good knowledge about preventing the disease. In contrast, Duruk et al. [1] reported a low score of 12.36%, and Cagetti et al. [10] reported a score of 74.5% with a surgical mask. Most of the participants had good knowledge (PGs: 88%, F: 89%, PPs: 95%) about the availability of a vaccine, which is similar to the study in China by Zhong et al. [18], with a score of 94%. In contrast, Singh Gambhir et al. [15] reported 42% of participants had sufficient knowledge regarding vaccines to prevent COVID-19.

These mainly included “fear of family getting infected,” perceptions towards the possibility of vaccine availability in the future, and support from the government institutions. Higher scores were obtained among all the participants for questions related to the transmission of the infection (PG-90%, F-92%, and PP-91%), and pediatric dentists expected to have adequate knowledge (PG-92%, F-97%, and PP-96%). Factors such as fear of transmission from infected patients, trending news on social media about the disease characteristics, and mortality rate might be the reason for good knowledge scores [19,20] There is still no definitive treatment and the prolonged incubation period further enhances the anxiety among pediatric dentists [9]. These findings are in agreement with an Austrian study performed among pediatric dentists [21]. It implies all the participants being very proactive in understanding the virulence of infection, the number of cases reaching the peak, projected in the social media, and implementing all efforts to prevent the spread of disease [22,23,24].

The majority of the participants had very little support from government institutions and dental practice guidelines available so far. The scores for the availability of support from government institutions were least in private practitioners (33%), with the faculty nearing a similar range (36%), followed by slightly higher scores among postgraduate residents (50%). These scores depict the rising concerns, especially among the private practitioners, due to fear of financial burden, fear of becoming infected, transmission to family members, and rising costs of treatment [20]. The least score was reported for the availability of professional guidelines among private practitioners (28%), followed by slightly higher but not satisfactory scores among faculty (40%) and private practitioners (44%). These results show the necessity to propose standard guidelines for dental practice before the pandemic outbreak. A recent study from China [25] performed with 2699 orthodontists suggested that COVID-19-related training programs necessitate enhancing knowledge and preparedness. A recent study used the same questionnaire tool [24] found adequate knowledge and perceptions scores among dental specialists from various clinical specialties. In this study, only 102 pediatric dentists were participated and the achieved good knowledge and perception scores with 9.2 ± 1.2 and 5.5 ± 1.6 mean scores. These finding are in agreement with the present study. The authors also opined that adequate and suitable protection should also be provided to ensure safety and reduce the psychological burden. However, the results cannot be applied to the present study as the Chinese study [25] was conducted among the orthodontists.

The key information source for knowledge on the infection and preventive measures was mainly from health authorities, compared to social media (*p* < 0.05). Many health care organizations such as the Centre for Disease Control (CDC) and the World Health Organization (WHO), and American Dental Association (ADA) have made tremendous efforts to educate health care professionals about virulence, modes of transmission, and recommended carrying out only emergency treatments [5,18,19,23,24]. In the present study, knowledge means the source of information influenced scores, and statistical significance was found among these two parameters. It is because of ease of access in the modern digital era that many social media platforms, such as Google, Facebook, WhatsApp, YouTube, and news channels have become the primary source of information. Health authorities were found to be successful in educating healthcare professionals, followed by social media [20,26] A recent population-based study from Saudi Arabia [27] reported that the ministry of health website was frequently used to learn information regarding COVID-19. Aldhuwayhi et al. [24] reported that health authorities and social media played a significant role in providing information regarding COVID-19. Nonetheless, in the present study, the participants opined that health authorities played a significant role in delivering updates on COVID-19. Kentikelenis and Seabrooke [28] reported that policymakers are crucial in preparedness during and post COVID-19. A Taiwanese study [29] reported that most of the participants depend on the internet to attain knowledge regarding COVID-19. A Jordanian [30] study reported that scientific articles and websites were commonly utilized to gain information regarding COVID-19 among medical students. In the present study, pediatric dentist’s knowledge scores were associated with age and source of information, while perception scores were associated with age and gender. There was no evidence supporting the association among these paraments. Nonetheless, the authors opine there is a need to establish the relation between the source information and influence on knowledge scores regarding COVID-19. A multinational study [19] also found a correlation between knowledge means scores and qualification of study participants. These findings are not comparable with the present study, where the study participants were only pediatric dentists.

A recent Italian study endorses the promotion of food education and healthy dietary style during this pandemic [31]. During this pandemic outbreak, telemedicine in pediatric dental clinics has also been recommended [32]. A recent study [33] from Saudi Arabia also opined that the majority of dental undergraduate students showed interest during this pandemic. An Austrian study [34] conducted among the dental students also finds imperative knowledge regarding COVID-19, and the authors found a lacuna concerning infection and hygiene control. However, the present study was performed among the pediatric dentist; hence, these findings from Saudi Arabian [33] study and the Australia [34] were not comparable. Another collaborative study [24] from India involving all dental clinical specialists from India also found a good knowledge and perceptions regarding COVID-19 using the same questionnaire. However, these results are also not comparable with the presents study.

An Australian study [35], a Polish study [36], and a German study [37] also evaluated knowledge scores among dentists. All the studies found sufficient knowledge regarding COVID-19. Nonetheless, the present study focused only on Pediatric dentists; henceforth, findings were not comparable. An Austrian study reported that all the pediatric dentists have sufficient knowledge of COVID-19, and most of them were accessible for emergency dental visits [21]. It is the only study performed among pediatric dentists regarding their knowledge of COVID-19. Both the studies achieved excellent scores regarding COVID-19 questions asked to the participants, and however, the questionnaire was different. Social media has become very popular for the survey, and the recent outbreak of COVID-19 has been witnessed [38]. Polish study [36] used major groups on Facebook Saudi Arabian study [39] used Twitter and WhatsApp, and multi-notational study from the Middle East used [40] social media outlets (e.g., Facebook and Twitter). Similarly, in the present study, social media in WhatsApp was used to send the survey to the participants. Wolf et al. [37] survey reported that COVID-19 changed the dental practice drastically. Subsequently, an Australian study reported that [35] holistic training is mandatory for successfully managing patients in the dental operatory during the COVID-19 outbreak. It has also been reported that many changes were evident in the dental operator, and it is also time to adopt the new strategies and inflectional protocols in the dental operatory [41,42]. A German study [43] reported that an additional measure includes compound daily patient care and patient time required with established infection control protocols are required in the dental operatory. An American study [44] reported that it is imperative to maintain social distancing and changes of personal protection equipment for every patient. In the present study, more than 80% of the pediatric dentist felt the use of PPE and social distancing is compulsory to avoid cross-contamination of COVID-19.

The findings from the present study may not be generalized and, however, could be used as a reference for further studies planned among pediatric dentists regarding COVID-19. The participants from WhatsApp groups responded to the present study questionnaire and no control over the response from the participants. The participants’ country was not considered, and the majority of the participants and this is one of the study’s limitations. Only 300 pediatric dentists participated in the study and 9 (3%) were excluded from the analysis due to incomplete responses to the questionnaire. These are possible limitations for the present study; however, being the first to gather knowledge and perceptions data among pediatric dentists can be one of the strengths of the study. The study also evaluated the source of information utilized by pediatric dentists, and the study showed health authorities were utilized more than social media. The parameters included in the questionnaire are excellent predictors of perceptions of pediatric dentists towards the pandemic. The significant outcomes of the study are good knowledge among all the participants about the virulence of the disease, preventive measures, concerns related to the availability of the vaccine, support from government institutions, fear of becoming infected and transmission to family members, and the need for professional guidelines to open dental practices post-pandemic.

## 5. Conclusions

The study reveals that pediatric dentists had sufficient knowledge regarding COVID-19 symptoms, infection control, and transmission mode and were aware of all the precautionary measures. The pediatric dentists’ age, occupation, and source of information influenced knowledge regarding COVID-19, whereas perceptions were influenced by the age and gender of the participants. Health authorities successfully educated pediatric dentists than the social media.

## Figures and Tables

**Table 1:** Comparison of the source of information utilized for COVID-19 among the pediatric dentist.

Source of Information	Postgraduate Students	Faculty	Private Practitioners	Total	*p*-Value
Social media	20 (38.4%)	50 (36.7%)	32 (30.1%)	102 (35.0%)	0.003 *
Health authority	25 (48.0%)	69 (50.7%)	38 (36.8%)	132 (45.3%)	
Both	7 (13.4%)	17 (12.5%)	33 (32.0%)	57 (19.5%)	
Total	52	136	103	291	

* There is significance at the 0.05 level using the chi-square test.

**Table 2:** Comparison of sources of information based on overall mean scores achieved by pediatric dentists.

Source	N	Mean	Std. Deviation	95% Confidence Interval for Mean		Minimum	Maximum	*p*-Value
				Lower Bound	Upper Bound			
Social media	102	8.89	1.41	8.61	9.16	2.00	10.00	0.001 *
Health authority	132	9.31	0.83	9.16	9.45	7.00	10.00	
Both	57	9.66	0.54	9.52	9.81	8.00	10.00	
Total	291	9.23	1.07	9.10	9.35	2.00	10.00	

* The mean difference is significant at the 0.05 level using ANOVA.

**Table 3:** Details of mean values of pediatric dentists based on occupations.

Characteristic/Value		Total	Postgraduate Students	Faculty	Private Practitioners	*p*-Value
**Knowledge**	Range of scores achieved (maximum 10)	2–10	2–10	2–10	5–10	
	Mean ± SD	9.21 ± 1.09	9.21 ± 1.09	8.96 ± 1.37	9.41 ± 0.83	
	Cut-off point	8	8	8	8	
	High/Good knowledge scores *n* (%)	73.1	73.1	80.1	90.3	0.018 *
	Low/Poor knowledge scores *n* (%)	19.9	19.9	26.9	9.7	
**Perceptions**	Range of scores achieved (maximum 10)	0–8	0–8	0–8	1–8	
	Mean ± SD	5.6 ± 1.6	5.6 ± 1.6	5.4 ± 1.6	5.7 ± 1.3	
	Cut-off point	6	6	6	6	
	High/Good knowledge scores *n* (%)	71	73	70	70	0.900 ^NS^
	Low/Poor knowledge scores *n* (%)	29	27	30	30	

SD—Standard deviation; *p*-value is calculated using the chi-square test; * significance *p* < 0.05; NS = Non significant.

**Table 4:** The multivariate linear regression analysis used the association between demographic variables and the pediatric dentists’ knowledge and perception scores.

Outcome	Predictor	Coefficients	95.0% Confidence Interval		*p* Value
			Lower Bound	Upper Bound	
**Knowledge**	Occupation (Postgraduate students)	ref	ref	ref	
	Occupation (Faculty)	0.566	0.197	0.935	0.003 *
	Occupation (Private Practitioners)	0.640	0.263	1.017	0.001 *
	Age (Years)	−0.146	−0.224	−0.068	0.000 *
	Gender (Female)	ref	ref	ref	
	Gender (Male)	0.052	−0.201	0.304	0.69 ^NS^
	Source of information about coronavirus (COVID-19)? (Social media)	ref	ref	ref	
	Source of information about coronavirus (COVID-19)? (Health authorities)	0.433	0.169	0.698	0.001*
	Source of information about coronavirus (COVID-19)? (Health authorities and social media)	0.728	0.390	1.065	0.000*
**Perceptions**	Occupation (Postgraduate students)	ref	ref	ref	
	Occupation (Faculty)	0.527	−0.010	1.064	0.06 ^NS^
	Occupation (Private practitioners)	0.679	0.130	1.228	0.01 *
	Age (Years)	−0.11	−0.21	−0.01	0.03 *
	Gender (Female)	ref	ref	ref	ref
	Gender (Male)	0.747	0.379	1.115	0.000 *
	Source of information about coronavirus (COVID-19)? (Social media)	ref	ref	ref	ref
	Source of information about coronavirus (COVID-19)? (Health authorities)	0.128	−0.257	0.512	0.514 ^NS^
	Source of information about coronavirus (COVID-19)? (Health authorities and social media)	0.240	−0.251	0.731	0.337 ^NS^

* ref—Reference; Statistically significant (*p* < 0.05); NS = Non significant.

## Data Availability

The raw data supporting the conclusions of this article will be made available by the authors, without undue reservation.

## Supplementary Materials

The following are available online at https://www.mdpi.com/article/10.3390/ijerph19010209/s1, Questionnaire-Form.
