# Supplementary material for: Knowledge and Perceptions Regarding Coronavirus (COVID-19) among Pediatric Dentists during Lockdown Period
Source: Int J Environ Res Public Health. 2021 Dec 25;19(1):209. doi: 10.3390/ijerph19010209 (PMC8750425; doi:10.3390/ijerph19010209)
Supplement: Supplementary file 1 [file ijerph-19-00209-s001.zip › ijerph-1510674-supplementary.pdf]

# Questionnaire- Form

Socio-demographic details:
1. Gender: Male/Female
2. Age(years): 19-25; 26-30; 31-35; 36-40; 41-45; 46-50; >50
3. Occupation: Faculty / Private Practitioners/ Postgradaute students
4. Source of Information:
   Source of Information: 1. social media- whatsapp, youtube, telegram, Facebook, Twitter, and Snapchat., etc; 2. Health authorities- WHO, CDC, ADA, university authorities, Local and international societies; and Both
5. Do you have sufficient knowledge about COVID-19?- Yes/ No/ Maybe

Knowledge:

| Questions | Response |
|---|---|
| 1. Do you know that COVID-19 is a viral infection? | Yes/No |
| 2. Do you know that COVID-19 is fatal? | Yes/No |
| 3. Do you know that COVID-19 is transmitted by close contact with an infected person? | Yes/No |
| 4. Do you know that fever, cough, and shortness of breath are symptoms of COVID-19? | Yes/No |
| 5. Do you know that the incubation period is COVID-19 is 2 weeks? | Yes/No |
| 6. Do you think the vaccine is available in markets? | Yes/No |
| 7. Do you think washing hands with soap and water or the use of sanitizer can help in the prevention of COVID-19 transmission? | Yes/No |
| 8. Do you know dentists are at a higher risk of COVID-19? | Yes/No |
| 9. Do you think patients with underlying chronic diseases are at a higher risk of COVID-19? | Yes/No |
| 10. Do you know that the use of N-95 can help in avoiding COVID-19? | Yes/No |

Perceptions:

| Questions | Response |
|---|---|
| 1. Are you worried one of your family members may get COVID-19? | Yes/No |
| 2. Transmission of COVID-19 can be prevented by using standard and isolation precautions given by WHO, CDC, ADA, etc? | Yes/No |
| 3. The prevalence of COVID-19 can be reduced by the active participation of health care workers in the hospital infection control program? | Yes/No |
| 4. If a COVID-19 vaccine is available, would you have it? | Yes/No |
| 5. Do you think dental treatment should be provided to patients with COVID-19 positive? | Yes/No |
| 6. Dentists must avail themselves of all information about the COVID-19? | Yes/No |
| 7. Is the available information about COVID-19 in your professional society sufficient? | Yes/No |
| 8. Are the government institutions able to control the pandemic? | Yes/No |
